# Prepulse Inhibition of the Auditory Startle Reflex Assessment as a Hallmark of Brainstem Sensorimotor Gating Mechanisms

**DOI:** 10.3390/brainsci10090639

**Published:** 2020-09-16

**Authors:** Ricardo Gómez-Nieto, Sebastián Hormigo, Dolores E. López

**Affiliations:** 1 Institute of Neurosciences of Castilla y León, University of Salamanca, 37007 Salamanca, Spain; richard@usal.es (R.G.-N.); sebifisio@gmail.com (S.H.); 2 Institute Biomedical Research of Salamanca, University Hospital of Salamanca, 37007 Salamanca, Spain; 3 Department of Cell Biology and Pathology, University of Salamanca, 37008 Salamanca, Spain; 4 Department of Neuroscience, University of Connecticut Health Center, Farmington, CT 06030, USA

**Keywords:** attentional modulations, neuroplasticity, PPI

## Abstract

When a low-salience stimulus of any type of sensory modality—auditory, visual, tactile—immediately precedes an unexpected startle-like stimulus, such as the acoustic startle reflex, the startle motor reaction becomes less pronounced or is even abolished. This phenomenon is known as prepulse inhibition (PPI), and it provides a quantitative measure of central processing by filtering out irrelevant stimuli. As PPI implies plasticity of a reflex and is related to automatic or attentional processes, depending on the interstimulus intervals, this behavioral paradigm might be considered a potential marker of short- and long-term plasticity. Assessment of PPI is directly related to the examination of neural sensorimotor gating mechanisms, which are plastic-adaptive operations for preventing overstimulation and helping the brain to focus on a specific stimulus among other distracters. Despite their obvious importance in normal brain activity, little is known about the intimate physiology, circuitry, and neurochemistry of sensorimotor gating mechanisms. In this work, we extensively review the current literature focusing on studies that used state-of-the-art techniques to interrogate the neuroanatomy, connectomics, neurotransmitter-receptor functions, and sex-derived differences in the PPI process, and how we can harness it as biological marker in neurological and psychiatric pathology.

## 1. Characteristics and Functional Implications of the Acoustic Startle Reflex

The acoustic startle reflex (ASR), a survival mechanism of alarm, rapidly alerts and arouses organisms to a sudden loud auditory stimulus. Behaviorally, the ASR involves a rapid and sequential activation of muscles along the length of the body as well as an autonomic physiological response [1]. In mammals, the most effective ASR is triggered by high-intensity sounds that exceed 80 dB and white noises are more effective than pure tones. An important factor in the elicitation of the ASR is the sudden onset of the stimulus but also its duration. The ASR is considered a defensive reaction to an unexpected sensory event that interrupts ongoing behavior and prepares the individual against a potential threat. This reaction activates a defensive stance to prevent injury and alerts the person or animal to initiate escape behaviors. In humans, the ASR involves a fast and involuntary flexor muscle contraction (flinch) with electromyographic responses of just 6–10 ms in latency.

This reflex is intimately related to another innate reaction exhibited by humans and nonhuman primates in early developmental stages [2] called the Moro reflex. The participation of the auditory and vestibular systems in this reflex is implied since it is triggered by a sudden loud noise or abrupt changes in head position. The Moro reflex is normally characterized by highly stereotyped movement patterns consisting of an embracing posture of the arms and flexion of the legs. The Moro reflex typically disappears at approximately 4 months postnatal and is replaced by a generalized contraction of facial and limb muscles that follows a rostrocaudal pattern [3], the ASR.

In small mammals, the ASR is manifested as a whole-body flinch and/or leap, directly proportional to the magnitude of skeletal muscle contraction. The ASR displays a short duration and latency as evidenced by electromyographic measurements of just 6 and 8 ms in the neck and hindleg muscles of the rat, respectively [4,5].

Decreased ASR responses related to aging have been well reported in both animal and human studies [6,7]. This hyporeactivity is not due to age-related hearing deficits but could be explained by brainstem processing delays that limit an individual’s ability to rapidly adjust to the environment [6,8].

The ASR can be easily assessed using electromyographic recording in humans and large animals, and whole-body ballistic movements in smaller animals (rodents) using a startle response system that consists of a piezoelectric accelerometer mounted under a platform that detects the corresponding startle responses [9,10]. In humans, surface electromyographic activity has been recorded from various muscle groups throughout the body, showing evoked response differences between them.

The most common muscles used for ASR measurements in humans, due to homogeneous and consistent electromyographic responses, are the facial, orbicularis oculi, and mentalis [8]. Among the most widely measured parameters are the amplitude (peak of startle response) and latency (time from stimulus onset to the peak amplitude of the ASR). Another important parameter is the probability of ASR occurrence in relation to the total number of trials. A recent paper reviewed and standardized the ASR measurement methodology in experimental animals [11].

There is controversy in the literature about sex differences in the ASR depending on the species tested. In the rat, the ASR amplitude is greater in males than in females [12], while other authors indicate that the ASR in humans is significantly smaller in men versus women [13]. Additionally, several authors point out that the magnitude of the ASR is not affected by sex or the phase of the estrous cycle [14]. Despite these contradictory results, it seems that variations in blood estrogen levels during the menstrual cycle might contribute to ASR variance through dopaminergic mechanisms in the brain. Thus, ovariectomized rats have persistent alterations in dopamine-mediated effects on ASR, and these alterations can be partly corrected with estradiol replacement [14].

## 2. Acoustic Startle Modulations

The ASR can be modified quantitatively or qualitatively by several natural and experimental conditions, indicating the individual’s ability to adjust responses to specific external and internal conditions. The ASR and its modulations, which are easily tested in humans and rodents, are sensitive to a variety of experimental approaches that consolidate these behavioral paradigms as essential research tools for studying brain mechanisms such as learning, memory, emotions, sensory gating, and movement control as well as drug treatments and, in humans, neuropsychiatric disorders (see below). The most relevant ASR modulations are briefly described in the following paragraphs.

### 2.1. Fear Potentiation of the ASR

The potentiation of the ASR by conditioned fear is a behavioral paradigm in which the amplitude of the ASR is enhanced in the presence of a conditioned neutral stimulus (light or tone) that was previously paired with an aversive unconditioned stimulus, for example, a foot shock [15]. The auditory fear-potentiated startle has been used to study Pavlovian fear conditioning, an important model in the study of the neurobiology of normal and pathological fear. This ASR modulation, based on (classical) conditioning processes, implies that the ASR might be a crucial part, and occasionally the triggering factor of fear and panic attacks that last longer than the reflex itself and can lead to the blocking of an individual’s reactivity.

Interestingly, the ASR is attenuated if elicited in a pleasant emotional context, for example, in the presence of a conditioned stimulus predicting reward [16,17].

### 2.2. Sensitization and Habituation of the ASR

According to the dual-process theory of nonassociative learning, a behavioral response during repetitive stimulation is influenced by two processes, sensitization and habituation. Thus, when a repetitive stimulus is presented to the subject, the sensitization of the ASR is the increase in ASR amplitude, whereas the habituation is the decrease. Both processes occur and develop independently of one another but interact to yield the final response output. Higher intensity levels of sound tend to govern sensitization [18]. That is, according to the stimulus intensity level, the stimulus can become a relevant and aversive event that has a sensitizing effect on the subsequent startle reflexes. Although repetitive stimulation has both habituating and sensitizing effects on the amplitude of the ASR, habituation dominates the course of amplitude, and sensitization dominates the course of latency [19]. Habituation of the ASR can be short-term (stimulus repetition within-session) or long-term (stimulus repetition between sessions), depending on presentation and the interval between startling stimuli.

The habituation of the ASR is not derived from physiological fatigue of the receptors or by adaptation events [19], and shows sex- and age-related differences. For instance, young rats exhibit reduced short-term habituation compared to older rats [20]. Additionally, young male rats exhibit reduced long-term habituation compared with older male rats, whereas older female rats habituated more slowly than older male rats. Along the same line, sensitization occurs more consistently in younger rats [20].

Recent studies in humans support a relationship of individual differences in personality with habituation in the ASR [21]. Thus, higher levels of neuroticism are related to faster habituation, whereas higher levels of aggressiveness are related to slower habituation.

### 2.3. Drugs Affecting the ASR

There are many drugs that have direct effects on the ASR. The most well-known drugs are the dopaminergic agonists that increase ASR amplitude and shorten its latency. For example, the direct dopaminergic agonists, bromocriptine and apomorphine [22,23], and the indirect dopaminergic agonists, amphetamine and cocaine [24,25], induce and intensify the startle response. In addition, serotonin releasers, such as “ecstasy” (3,4-methylenedioxymethamphetamine or MDMA), induce changes in the ASR and its modulations [26]. Other sedative/anxiolytic drugs, such as diazepam and clonidine, reduce the ASR amplitude and increase the ASR latency [27]. Alcohol and tobacco affect the ASR differently. Alcohol decreases the startle magnitude, and nicotine decreases startle latency without modifying the amplitude [28]. The ASR is also considered a good marker of anxiety disorders, showing that ASR amplitude increases with anxiety and stress [29]. A recent study reports an increase in ASR amplitude as an effect of prenatal stress [30].

### 2.4. Prepulse Inhibition of the ASR

The ASR can be reduced when a strong acoustic startling stimulus (pulse) is immediately preceded by a weak non-startling stimulus (acoustic, visual, or tactile)—a paradigm called prepulse inhibition (PPI). The amount of PPI is widely used as a quantitative measure of sensorimotor gating and a filtering mechanism of the central nervous system to prevent sensory information overload, facilitating selective attention and ensuring normal information processing. PPI provides a valuable method for investigating the principles of reflex modulation in humans and experimental animals [31,32]. It occurs on the first exposure to the prepulse and pulse stimuli without exhibiting habituation or extinction over trials, and therefore, PPI is not a form of conditioning. Most studies using the PPI paradigm calculate the percentage of the magnitude of the PPI for each respective prepulse intensity according to the following formula: % prepulse inhibition = (100 − (100 × startle amplitude on prepulse followed by pulse trial)/(startle amplitude on pulse trial alone)). PPI values are influenced by physical parameters of the prepulse and pulse stimuli such as intensity, duration, frequency and interstimulus intervals (time between the prepulse and the startling pulse). PPI increases with both prepulse intensity [33] and duration [32] and is also sensitive to the frequency difference between the prepulse and background tones [34]. In experimental animals, PPI occurs with interstimulus intervals of 20–500 ms, or even at shorter intervals. In humans, the prepulse inhibition paradigm uses combinations of interstimulus intervals ranging from short interstimulus intervals (between 30 and 300 ms), in which case the paradigm process is referred as PPI, and long interstimulus intervals (more than 500 ms), which in this case are named prepulse facilitation (PPF) [35].

It is clear that gender contributes to the variability in PPI measures. Thus, PPI values are higher in males than in females in both humans [36] and experimental animals [12]. Furthermore, human females show varying PPI across the menstrual cycle with the highest levels in the follicular phase and the lowest levels in the luteal phase [37]. However, there is no clear consensus on the effects of age on PPI. Some authors suggest that there is no effect of age on PPI in adult mice [38] and humans [39]. By contrast, several studies describe that PPI is reduced in older rats [7] and aged humans [40]. In humans, PPI is probably not fully functional until 8–10 years old [41], and PPI shows an inverted U-shaped function depending on age, resulting in the highest PPI values at intermediate ages [40]. A deficit in sensory gating, as measured by PPI, could be the cause of selective attention deficits and increased distractibility in the elderly. Unpublished data from our research group agree with Ellwanger et al. (2003) [40], suggesting that only groups of extreme ages in humans (<20 and >73 years old) exhibit no differences in PPI between them. PPI can be modified with pharmacological treatments [42], stress, nicotine and caffeine consumption and abstinence. Thus, PPI increases with nicotine consumption [32] and decreases with nicotine abstinence [43]. The effects of caffeine on PPI are complex and depend on the dose. Changes in the environment such as early maternal separation or social isolation can affect PPI, as shown in rats [44].

## 3. Research and Clinical Applications

The ASR and its modulations can be easily tested across species and are often used as an easy, affordable, non-invasive and objective tool for assessment of behavioral plasticity mechanisms of sensorimotor gating, emotional status, as well as morphofunctional aspects of the normal and pathological nervous system. These behavioral paradigms are a reliable and robust quantitative phenotype across species, and therefore they are considered relatively stable neurobiological markers [31,45]. Since the ASR is influenced by the emotional and attentional context, the modulations of the ASR have been used in psychology as a measure of emotional changes (fear, aversion and pleasure).

The sustained advances in the study of ASR modulations in experimental animals resulted in the development of equivalent models for understanding similar conditions in humans, becoming a research tool for drug testing. Thus, the enhanced ASR leads to a panic state in the experimental animal that is equivalent to the state of psychological anxiety in humans [46], and hence it is used to study the effects of drugs that increase (e.g., yohimbine) or reduce (e g. benzodiazepines) the ASR. The ASR modulations open the possibility to use these behavioral paradigms for the study of various neuropsychiatric disorders [3]. Adolescents with behavioral disorders and emotional insensitivity, related to antisocial behavior, show deficits in fear conditioning and a deficit in the startle response [47]. In addition, stress and drug addiction resulted in abnormal startle reflex [48]. Patients with neurological disorders such as hyperekplexia [49], reflex myoclonus [50] and Parkinson [51] exhibited exacerbated ASR amplitudes. Other diseases, such as the idiopathic restless legs syndrome, resulted in a significant reduction in ASR latency [52]. The startle reflex has also been considered as a possible tool for early detection of motor dysfunctions and neurological damage [53], as well as to evaluate the integrity of the hearing system. In fact, the absence of the Moro reflex may indicate the presence of a lesion or disease.

PPI has been used as a technique for assessing ototoxicity [54]. As already referred to in the previous section, numerous studies have demonstrated PPI deficits in schizophrenic and schizotypal personality-disordered patients, as well as in rats treated with drugs exerting psychotic effects [55,56]. PPI measurement could become a reliable diagnostic tool for these neuropsychiatric disorders, because it uses an objective biological signal that might help to establish an early diagnosis, provide an index of the severity of pathology and serve to test the efficiency of treatment in an objective way [32].

In summary, PPI measurement could become a reliable tool as an endophenotype for genomic studies and a biomarker for healthy brain circuitry, which may predict sensitivity to psychotherapeutics [32,55].

## 4. Prepulse Inhibition as an Indicator of Neural Plasticity

Since PPI protects the early processing of the prepulse signal from startling interferences by regulating the motor system and/or the premotor system, it has been generally recognized as an operational measure of sensorimotor gating [57,58].

Modifications of the PPI are very diverse and may be caused by neurological [59] or psychiatric diseases characterized by anxiety symptoms [60,61], behavioral states (such as emotional context or a stressor) [62,63,64], hormonal levels [37,65,66], and even adaptations derived from particularities of some professions, as occurs in the case of athletes [67]. All reflect various processes of neural plasticity, and it is striking that their sensitivity to sensory events happen a few milliseconds before the startle-eliciting stimulus. All of this makes PPI a reliable and robust quantitative phenotype across species, and it may serve as a relatively stable neurobiological biomarker for various pathologies or attitudinal characteristics [42,45].

It has been postulated that the startle response may interrupt information processing of the prepulse stimulus (the so-called interruption hypothesis) and also that an inhibitory system is activated by a prepulse in order to decrease that interruption (the so-called protection hypothesis). Any process that favors the startle response would facilitate the interruption of the information processing, and the opposite would occur with the mechanisms that increase the PPI. This hypothesis, formulated by Graham (1975) [68] but better known as Blumenthal hypothesis [69], provides a basic scheme on which other processes are superimposed (selective attention, accessory stimuli, alert signals, etc.), which can displace the system in one way or the other.

Among all the PPI modifications, the following deserve to be highlighted since they deal with processes related to neural plasticity:

### 4.1. Attentional Modulations of PPI

PPI protects the early processing of the prepulse signal from startling interferences by regulating the motor system and/or the premotor system. We can distinguish two components of the PPI, one involuntary (an automatic process at the preattentive stage) and another modulated by attentional responses, indicating modulation by higher-order cognitive processes [44]. The deeper layers of the superior colliculus play a role in mediating the attentional enhancements of PPI, probably through both receiving top-down signals from certain forebrain structures and modulating the midbrain representations of prepulse signals [70]. The participation of the thalamus in the attentional modulations of PPI becomes evident with adverse prestimuli, in which the amygdala [43], as well as the inferior colliculus [71], plays a key role in PPI enhancement. This has been confirmed in human fMRI experiments, in which a paradigm of attention to the prepulse indicates that thalamic areas are involved [72].

As mentioned above, when the prepulse-to-pulse intervals are longer than 500 ms, the phenomenon is known as prepulse facilitation (PPF) and reflects sensory enhancement and selective attention [73,74]. Several studies have shown that voluntarily directing attention toward some aspect of the prepulse affects both PPI and PPF. Thus, humans who attended to the prepulse exhibited larger PPI at interstimulus intervals of 120 ms compared to participants who ignored the prepulse (but not at shorter lead intervals such as 60 ms), and PPF is greater during the attended prepulse than during the ignored prepulse. Thus, passive PPI and PPF are primarily automatic processes, whereas attentional modulation involves controlled cognitive processing [75]. Facilitation of relevant stimuli (PPF) and inhibition of irrelevant stimuli (PPI) constitute separable aspects of selective attention and is differentially affected by age [40], gender [76], species [77] and interstimulus intervals [78]. In this last aspect, there is a contradiction since interstimulus intervals smaller than 15 ms also trigger an increase in startle amplitude, resulting in net response facilitation in rats [4]. There is general agreement that both PPI and PPF processes are two independent processes [79]; first, PPI and PPF were affected differently by the prepulse intensity. PPI increased as the prepulse intensity increased. The PPF, however, did not linearly depend on the prepulse intensity but first increased as prepulse intensity increased, followed by a decrease [77]. PPF is the result of temporal integration of neural activity within the startle pathway initiated first by the prepulse and then by the startle stimulus. In this case, Ison et al. (1973) [4] suggested that the PPF might be mediated by either an excitatory interaction between the sensory responses to the prepulse and to the startle stimulus or a process of motor preparation elicited by the prepulse. In any case, both processes reflect mechanisms of brain plasticity in response to stimuli of different intensities and times, which should undergo learning during repeated testing [77].

### 4.2. Gap-Prepulse Inhibition of the Acoustic Startle Reflex for Tinnitus Assessment

Tinnitus is the medical term for “hearing” noise in the ears when there is no external sound source. Tinnitus decreases when people with this disorder focus on activities that absorb them [80] and do not require signal processing in the auditory domain. Modulation of tinnitus awareness can fluctuate rapidly, suggesting either that the neural activity underlying tinnitus is dynamically altered or that its access to consciousness is gated by brain mechanisms that are sensitive to context or task demands, a clear example of neural plasticity.

A form of prepulse inhibition (PPI) of the ASR is a widely accepted method for detecting tinnitus in rodents [81,82], the so-called Gap-induced Inhibition of the Acoustic Startle (GPIAS). This method relies on a short gap in a continuous background noise or tone to provide a cue that inhibits the usual startle response following a loud sound [83]. The gap acts as a prepulse in reducing the startling response, but the same does not apply to animal tinnitus models [81,84]. It seems that tinnitus acts to fill the gap in the background noise, affecting the unconscious neural processing of GPIAS in the brainstem [85].

There is no general consensus on the use of GPIAS in humans to assess tinnitus [85]. The studies carried out so far are discordant among themselves and also from animal studies [86]. This was presumably due to the lack of knowledge of the basic startle circuit, which has been described only in rodents [9,87]. It is therefore increasingly important to improve understanding of the neuronal substrates underlying the ASR in humans in order to validate the tools used in experimental animals and turn them into more reliable and valuable diagnostic tools.

## 5. Neuronal Pathways of the Acoustic Startle Reflex and Its Prepulse Inhibition

**Figure 1:**
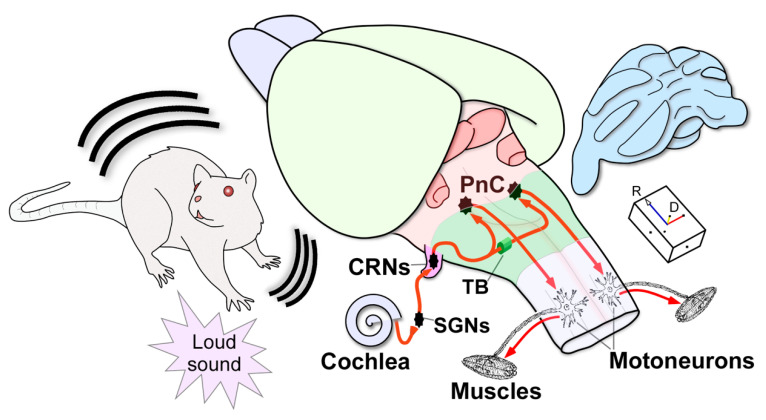
Three-dimensional (3D) schematic drawing of the primary acoustic startle circuit in the rat. Firstly, a sudden loud sound activates the sensory hair cells in the cochlea. Next, the spiral ganglion neurons (SGNs) innervate the cochlear root neurons (CRNs), which comprise the first relay in the brainstem, exhibiting a secure neuronal response with first-spike latencies of approximately 2.2 ms. Then, this short-latency input is quickly transmitted through bilateral projections via the trapezoid body (TB) to the giant neurons in the pontine reticular nucleus (PnC) that produce short-latencies of 5.2 ms. Finally, acoustically driven PnC neurons innervate motoneurons in the spinal cord to elicit the acoustic startle reflex with electromyographic responses of 6–10 ms. The arrowheads indicate the flow of neuronal information within the circuit. Projections from CRNs to other non-auditory nuclei, which are implicated in the full expression of the acoustic and pinna reflexes, are not depicted in this drawing. Abbreviations: CRNs, cochlear root neurons; D, dorsal; PnC, pontine reticular nucleus (caudal part); R: rostral; SGNs, spiral ganglion neurons; TB: trapezoid body.

The ASR is characterized by its short latency, and hence, it is widely accepted that a relatively simple pathway located in the brainstem mediates this reflex. The primary ASR circuit has been fully described in the rat (Figure 1). The cochlear root neurons (CRNs), true sentinels of the rodent auditory pathway, are the first brainstem neurons that receive direct input from the spiral ganglion cells in the cochlea [9,88]. CRNs innervate giant neurons in the caudal pontine reticular nucleus (PnC) of both hemispheres [87,89], which in turn project to facial, cranial and spinal motoneurons that rapidly activate muscle contractions [9,10]. At present, the ASR circuit is yet to be determined in other animals, although studies in cats have shown involvement of the brainstem reticular formation, especially the PnC [90]. In humans, the neuronal pathways of the ASR and its modulations are not known. It can be said, however, that the startle reaction is generated in the brainstem [91] because the startle reflex is observed in anencephalic infants [92]. Therefore, neurons in the reticular formation convey the output of the brainstem system, modulating and triggering startle across species. The neuronal pathways that modulate the ASR are more complex.

Since there is a high degree of homology between measures of PPI in rodents and humans [32], investigations of PPI-mediating pathways are critical for establishing new animal models for studying both cognitive features and neural bases of some neuropsychiatric disorders, which are characterized by PPI deficits. PPI is valuable for evaluating animal models of neuropsychiatric disorders that present with PPI deficits, “mapping” the corresponding neural substrates, and advancing the discovery and development of novel therapeutics. There are multiple pathways for mediating PPI [44]. Among these, Fendt et al. (2001) [93] suggested that auditory prepulses are processed via the ascending auditory pathway including the inferior colliculus (IC), which activates the superior colliculus (SC) that also receives input from other sensory modalities (auditory, visual and tactile). The anatomical connection between the SC and the pedunculopontine tegmental nucleus (PPTg) activates a cholinergic projection to the PnC that mediates the PPI. In contrast to the longstanding hypothetical view that PPI is mediated by cholinergic PPTg neurons, recent studies applying optogenetics in rats [94] or using selective cholinergic lesion in PPTg [95] have demonstrated that the non-cholinergic PPTg neurons mediate PPI, whereas cholinergic neurons of PPTg enhance startle and lead to prepulse facilitation [94]. This controversy should be explored further to clarify the presumed role of PPTg cholinergic neurons in the sensory gating mechanisms of the ASR.

Additionally, the substantia nigra contributes to the mediation of PPI via direct and indirect projections to PnC [44,96]. Behavioral positron emission tomography (PET)-imaging studies, showing the neural correlates and functional networks of PPI in awake rats [97,98], also include the cuneate nucleus (CuN) in the PPI mediation pathway. In support of this, the CuN sends a strong inhibitory projection to PnC [99], and Saitoh et al. (1987) [100] have reported that electrical stimulation of the CuN evokes PPI.

Nevertheless, this neuronal circuit does not explain all of the singularities of PPI such as the effectiveness of interstimulus intervals as short as 20 ms [101]. Recent studies support the existence of additional pathways that bypass some components of this long multimodal circuit to mediate fast auditory PPI via the cholinergic projection from the ventral nucleus of the trapezoid body (VNTB) to the CRNs [102,103,104] or the projections from the locus coeruleus to the CRNs, which could explain the sex differences in acoustic startle response and PPI [105].

**Figure 2:**
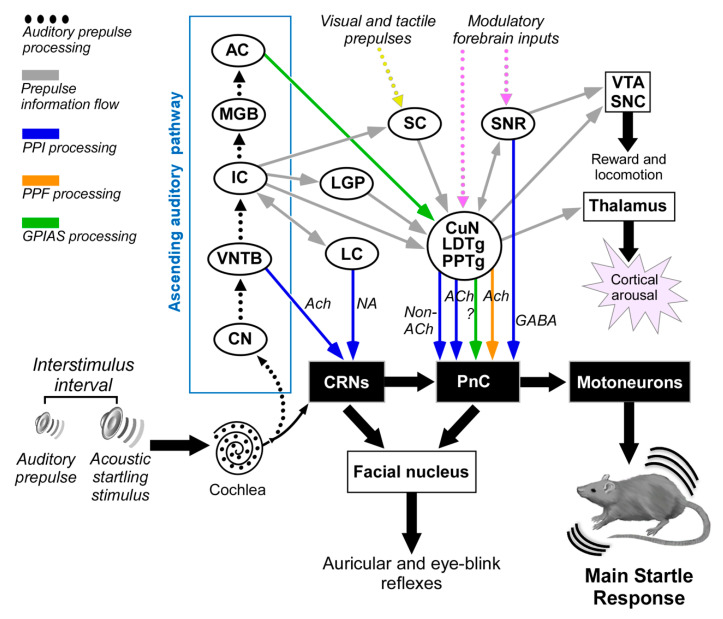
Multiple neuronal pathways for sensorimotor gating processes modulating the acoustic startle reflex in experimental animals. The auditory prepulse is processed through specific auditory nuclei in a serial hierarchical fashion and is then transmitted to many structures outside the auditory pathway. The auditory prepulse inhibition (depicted in blue arrows) involves the participation of VNTB-to-CRN projections that mediate fast acoustic inputs to reduce CRN responses at short interstimulus intervals, while the IC, SC, CuN, laterodorsal tegmental nucleus (LDTg), PPTg to PnC pathway mediates a slower pathway for auditory prepulse inhibition at long interstimulus intervals. Acetylcholine and other non-cholinergic neurotransmitters exert modulation on the CRNs and PnC. Notice that the IC might also bypass the SC via direct projections to PPTg. Furthermore, the GABAergic projection from the SNR to the PnC might be part of the PPI mediating pathway. The prepulse facilitation (depicted in orange arrow) implies cholinergic projections from PPTg to PnC. The gap-induced inhibition of the acoustic startle (GPIAS, depicted in green) involves the participation of the primary auditory cortex and PPTg. Tactile and visual prepulses are integrated into the PPI mediation circuit by some structures processing multi-modal cues. This connection diagram summarized data from the following studies: [85,93,104,105,106,110,111]. Abbreviations: AC: auditory cortex; CN: cochlear nucleus; CRNs: cochlear root neurons; CuN: cuneiform nucleus; IC: inferior colliculus; LC: locus coeruleus; LDTg: laterodorsal tegmental nucleus; LGP: lateral globus pallidus; MGB: medial geniculate body; PnC: caudal pontine reticular nucleus; PPTg: pedunculopontine tegmental nucleus; SC: superior colliculus; SNC: substantia nigra, pars compacta; SNR: substantia nigra, pars reticulata; VNTB: ventral nucleus of the trapezoid body; VTA: ventral tegmental area.

In sum, the existing reports and literature indicate that the existence of several parallel midbrain pathways that might act either jointly or separately for the PPI mediation seems very likely (Figure 2). The faster auditory PPI pathways include the VNTB-CRNs projection for short interstimulus intervals [10] and the IC-PPTg projection for long interstimulus intervals [106], as well as the slower multimodal PPI pathways that serially connect the IC, the SC, and the PPTg to the PnC [44,70,93,107]. According to Rohleder and coworkers [97,98], other nuclei are also involved in the rat PPI modulation or regulation, such as the nucleus accumbens, the habenula, the prelimbic cortex and the basolateral amygdala.

Finally, although GPIAS is a form of PPI, both differ in some of the temporal characteristics and the neural circuits involved in gap or prepulse inhibition. PPI is stable within a wide range of stimulus intervals, while GPIAS is more effective with shorter lead times [108] Furthermore, the primary auditory cortex (AC) seems to be decisive in GPIAS but not in PPI [109]. *c-Fos* induction experiments show that there is an increased activation of the lateral globus pallidus after PPI sessions and an increase in the *c-Fos* activation in the AC after GPIAS sessions. These results indicate that only the inhibition of the startle pulse by gaps triggers *c-Fos* induction in the AC [110].

Figure 2 summarizes the current knowledge of the PPI-mediating circuits based on experiments carried out on laboratory animals.

All the proposed human-related startle reflexes and PPI circuits are still pure speculation, since the basic circuit of the human ASR is currently unknown. However, behavioral, pharmacological and psychophysiological studies suggest the involvement of a complex neural network extending from brainstem nuclei to higher order cortical areas. The neuronal structures that regulate the PPI-mediating circuit include the limbic cortex, striatum and pallidum, as well as the pontine tegmentum (limbic “CSPP” circuitry) (for a review see Swerdlow et al., 2016) [55]. Additionally, other psychiatric pathologies, other than schizophrenia, which involve decreased PPI and are accompanied by brain disorders characterized by intrusive thoughts and images, sensations and movements, support the role of thalamic nuclei in the PPI modulator circuit, thus keeping limbic cortico-striato-pallido-thalamic (CSPT) circuitry in both the regulation of PPI and in the pathophysiology of these other brain disorders [112].

Several studies using functional magnetic resonance imaging to investigate the neural network underlying human PPI suggest a primary circuitry of sensorimotor gating in the pontine brain stem that interconnects with temporal, frontal and prefrontal cortices via the thalamus and striatum [113,114,115,116].

The apparent overlap in the neural substrates regulating PPI with those implicated in the pathophysiology of human brain disorders is part of the support for the etiological validity of animal models for impaired PPI in these disorders [55,114]. Finally, as pointed out by Swerdlow et al. (2016) in their review of 25 years of sensorimotor gating of the startle reflex [55], the fact that some PPI-regulatory neural mechanisms are conserved across species, from zebrafish (in which PPI is disrupted by apomorphine and restored by antipsychotics) [117], mice, rats, guinea pigs [111,118,119], pigs [120], lower primates [121] and higher primates [60], continues to make PPI an appealing measure for cross-species analyses of neural circuit connectivity.
